# The Potential Role of Motor Unit Number Estimation as an Additional Diagnostic and Prognostic Value in Canine Neurology

**DOI:** 10.3389/fvets.2015.00053

**Published:** 2015-11-10

**Authors:** Julia Kauder, Susanne Petri, Andrea Tipold, Veronika M. Stein

**Affiliations:** ^1^Department of Small Animal Medicine and Surgery, University of Veterinary Medicine Hannover, Hannover, Germany; ^2^Department of Neurology, Hannover Medical School, Hannover, Germany

**Keywords:** motor unit number estimation, compound muscle action potential, single motor unit potential, motor neuron disease, electrophysiology

## Abstract

Motor unit number estimation (MUNE) is an electrophysiological technique to assess the number of motor units innervating a single muscle or muscle group of interest. It may quantify axonal loss in any disease involving injury or degeneration of ventral horn cells or motor axons. Since MUNE has rarely been used in veterinary medicine, our study aimed to evaluate its potential role as an additional diagnostic and prognostic parameter in canine neurology. Therefore, we examined five healthy dogs and seven dogs suffering from diseases that necessitated general anesthesia for further diagnostics and treatment and that were not expected to interfere with the results of electrodiagnostic testing. By using the incremental technique to study MUNE in the cranial tibial muscle, we determined the number of motor units, the size of the compound muscle action potential, and the mean size of individual motor unit potentials of each dog as well as the mean values for each group. Moreover, we studied the correlation between these parameters. Taking the results into consideration, we addressed the difficulties and limitations of this technique. We, furthermore, pointed out possible fields of application for MUNE in canine neurology, and emphasized several aspects that future studies should focus on when applying MUNE to canine patients.

## Introduction

Neurological diseases play an essential role in veterinary medicine. Electrodiagnostic testing is already established as an integral part of the clinical evaluation of neurological canine patients. These examinations, such as electromyography (EMG) and nerve conduction studies (NCS), are performed routinely during the course of the diagnostic workup (1). Motor unit number estimation (MUNE), however, has rarely been applied in veterinary science. To the best of our knowledge, there are only two studies making use of this technique in dogs (2, 3). MUNE is an electrophysiological method for quantification of the number of motor units or axons innervating a single muscle or muscle group (4). The original MUNE method, described by McComas et al., is the incremental stimulation technique (5). Although various MUNE methods have been developed throughout the last decades, all of them share the same concept based on the original McComas technique. Assuming that the increase of the amplitude of the compound muscle action potential (CMAP) is due to a stepwise addition of single motor unit potentials (SMUPs), MUNE is obtained by calculation of a simple ratio of the maximal CMAP amplitude divided by the average SMUP (5, 6). The different MUNE techniques may solely be distinguished from one another by the way of acquisition of the sample of motor units, used to calculate the average SMUP (7).

There is extensive literature concerning the potential of MUNE in human patients and rodent models suffering from motor neuron disease such as amyotrophic lateral sclerosis (ALS). Mancuso and coworkers, studying transgenic mouse models of ALS, have demonstrated that MUNE can detect subclinical changes in motor unit number before onset of clinical signs (8). Others have shown that MUNE is even able to predict disease onset (9). Based on these studies, it has been concluded that this technique is not only capable of quantifying axonal loss in muscles of interest. It may, moreover, contribute to confirmation of the diagnosis, monitoring of disease progression, and response to treatment in diseases affecting the lower motor neuron (6, 10, 11). Taking into consideration that MUNE has hardly been used in veterinary medicine, the objective of this study is to determine whether it is generally applicable to canine patients. Focusing on the incremental technique, this study addresses its difficulties and limitations.

Furthermore, it intends to evaluate to what extent MUNE might be considered a reliable additional parameter concerning diagnosis and prognosis of canine neurologic patients.

## Materials and Methods

### Ethics Statement

All experiments were approved by ethical review, licensed by the Lower Saxony State Office for Consumer Protection and Food Safety (permit number: 33.19-42502-05-15A533) according to the German welfare act. The owners of the dogs of the heterogeneous group (see paragraph below) gave their written consent to the enrollment in our study.

### Canine Patients

This study included a heterogeneous and a homogeneous group of dogs. The heterogeneous group consisted of seven randomly chosen dogs presented at the Department of Small Animal Medicine and Surgery, University of Veterinary Medicine Hannover, Germany in order to undergo general anesthesia for further diagnostics and treatment of their diseases that were not expected to influence the results of electrodiagnostic testing. The electrophysiological examination was applied before or after the primary surgical or diagnostic intervention. The homogeneous group was formed by five healthy, purpose-bred Beagle dogs owned by the Department. Table 1 summarizes the different breeds, sexes, ages, body weights, and the diagnoses/reasons to undergo general anesthesia of both groups of dogs.

**Table 1:** **Overview of signalement of each dog**.

Dog number	Breed	Sex	Age (years)	Body weight (kg)	Diagnosis/reason to undergo general anesthesia
1	French Bulldog	Male	5	13	Low grade herniated disk C5-C6, MRI of cervical spine + head^a^
2	German Shepherd	Male	6	38	CT scan after total hip replacement on left pelvic limb^b^
3	Golden Retriever	Female	6	27	Ovariohysterectomy + unilateral mastectomy
4	Flat-Coated Retriever	Female	6	28	Peripheral brachial plexus tumor, MRI of left shoulder^c^
5	Airedale Terrier	Female	5	25	Ovariohysterectomy
6	Border Collie	Male	4	18	Traumatic radial paresis, electrodiagnostic testing^d^
7	German Hound (Bracke)	Male	3	26	Skin lump removal (thoracic wall)
8	Beagle	Female	2	11	MUNE
9	Beagle	Female	2	10	MUNE
10	Beagle	Female	3	10	MUNE
11	Beagle	Male	6	15	MUNE
12	Beagle	Male	2	12	MUNE

*MRI, magnet resonance imaging; CT, computed tomography*.

*^a^Normal proprioceptive placing and spinal reflexes in the hind limbs, hopping on the right pelvic limb slightly delayed*.

*^b^MUNE was performed on the right pelvic limb*.

*^c,d^No neurological deficits in the hind limbs*.

*^a,c,d^Even though these dogs did suffer from neurological diseases, we do believe that these diseases did not interfere with the results of our electrodiagnostic testing in the cranial tibial muscle. Therefore, the dogs were included in the study*.

### Anesthesia

The electrophysiological examination was performed under general anesthetic. Thus, the dogs initially received a premedication composed of Diazepam (0.5 mg/kg) and Levomethadone (0.2 mg/kg). Propofol (given to effect) was used for intubation and anesthesia induction, and Isoflurane in pure oxygen for maintaining anesthesia. Throughout the examination, all vital signs including rectal body temperature were constantly examined and recorded.

### Motor Unit Number Estimation

All electrodiagnostic studies were performed in a shielded room (Farraday cage) using Natus Keypoint Focus NT EMG equipment (Natus Europe GmbH, Planegg, Germany) with Keypoint.net 2.32 MUNE software and disposable monopolar needle electrodes commercially available from the manufacturer. The stimulation electrodes (Viasys disposable monopolar needle electrode, 50 mm × 26 G) were inserted percutaneously between the tuber ischiadicum and trochanter major in order to stimulate the sciatic nerve. Recordings (Natus disposable concentric needle electrode, 25 mm × 30G) were obtained from the cranial tibial muscle preferably on the right pelvic limb through standard amplifiers at a bandpass of 20 Hz–10 kHz. Reference electrodes (Spes medica disposable needle electrode, 13 mm × 33G) were placed subcutaneously close to the stimulation/recording electrodes. The ground electrode (Spes medica disposable needle electrode, 13 mm × 33G) was placed subcutaneously, axially at the level of Th1–Th3. In some cases, repositioning of the needles was necessary. Flexion of the tarsal joint upon stimulation indicated correct placement of the electrodes. In general, the estimated number of motor units innervating a target muscle or muscle group of interest can be determined by the following equation: MUNE = size of CMAP/average size of SMUP (11).

**Figure 1:**
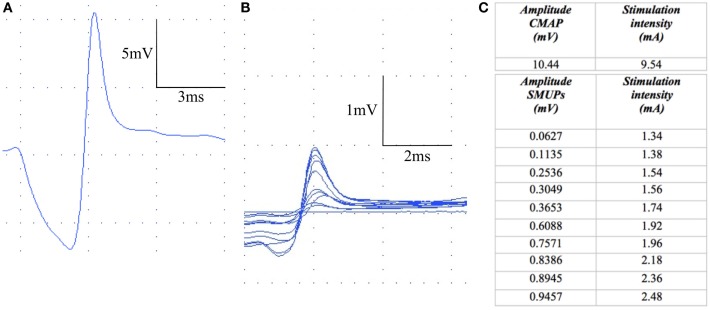
**The procedure of MUNE**. First, the CMAP is determined **(A)**. Then, a sample of 10 single motor unit potentials (SMUPs) is obtained **(B)** by constantly increasing the stimulus intensity **(C)**.

To obtain these two parameters, a version of the incremental stimulation technique described by McComas (5) was applied. To begin with, the maximal CMAP reflecting all motor units firing together (6) was evoked by supramaximal (7, 12) continuous stimulation (1 Hz, 0.1 ms monophasic stimuli). Subsequently, a small sample of motor units was determined from which the average size of a single motor unit action potential could be calculated. Therefore, the stimulus intensity starting from a subthreshold value was gradually increased until a small all-or-none response was evoked. This first increment represented the first motor unit being recruited (5, 10). The procedure was repeated 10 times by constantly increasing the intensity, and applying single pulse stimulation (Figures 1A–C). Thus, quantal increases in the response were recorded (9). By averaging the sizes of the 10 motor units, the mean size of a SMUP could be determined. The estimated number of motor units within the cranial tibial muscle was yielded by entering the data for the CMAP and the average SMUP into the equation mentioned above. For both CMAP and SMUPs, negative amplitude (baseline-peak amplitude) was measured. Due to a phenomenon termed alternation, the number of motor units in a muscle or muscle group may easily be overestimated (13). It refers to the fact that an incremental increase in the amplitude of the CMAP does not necessarily reflect the activation of another single motor unit but instead might be the result of an alternating activation of two different motor units already being recruited with overlapping thresholds. Hence, only increases >50 μV in the CMAP amplitude were accepted in order to ensure that an additional motor unit was recruited (8).

### Statistical Analysis

Statistical calculations of all electrophysiological test results were performed using GraphPad Prism software (GraphPad software inc., version 5.02, San Diego, CA, USA). All data were reported in the text as means ± SEM. The Mann–Whitney non-parametric test was used to assess the difference between the two groups after testing for normal distribution. Linear regression analyses were carried out for assessment of correlation between variables. *P* < 0.05 was considered to indicate a statistically significant difference.

## Results

**Figure 2:**
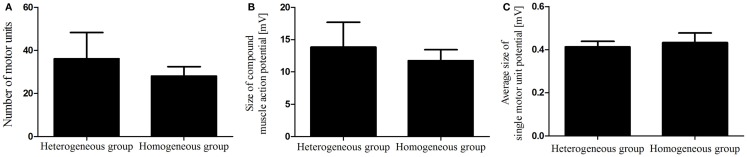
**Evaluation of functional motor units (A), CMAP (B), and mean SMUP (C) in both groups**. Values are mean ± SEM; *P < 0.05.

**Figure 3:**
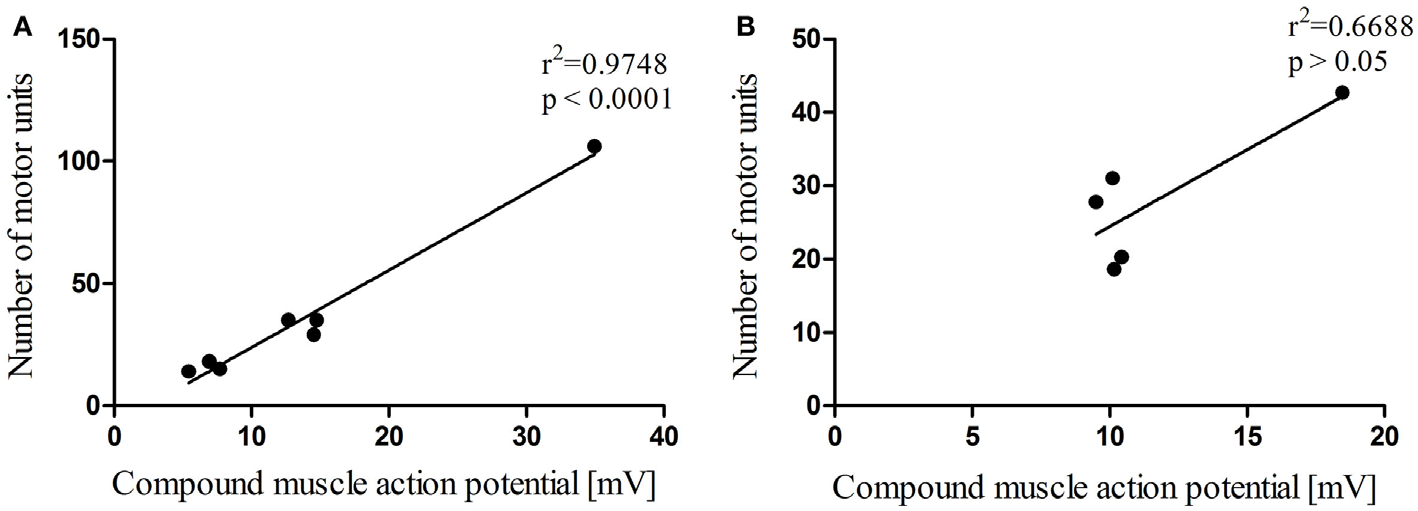
**Correlation analysis between the number of motor units and the CMAP**. The decrease in the number of motor units correlates to the reduction of the CMAP in the heterogeneous group **(A)**. No significant correlation was found in the homogeneous group **(B)**. Values are mean ± SEM; *P < 0.05.

**Figure 4:**
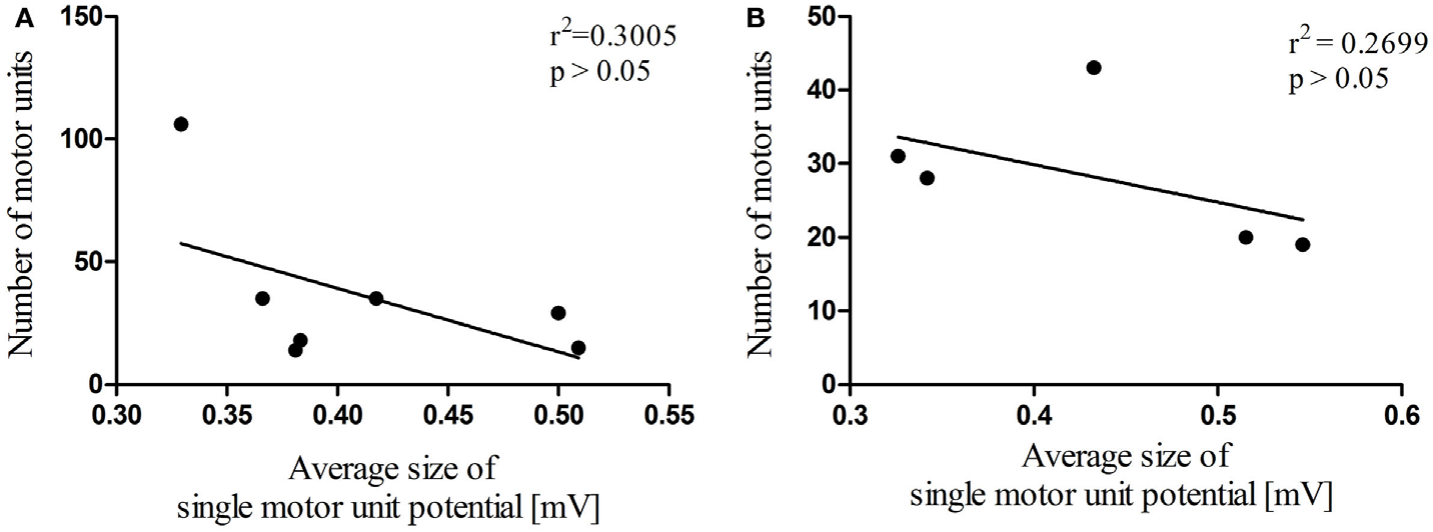
**Correlation analysis between the number of motor units and mean SMUP**. A significant correlation could neither be found in the heterogeneous group **(A)** nor in the homogeneous group **(B)** Values are mean ± SEM; *P < 0.05.

In total, 12 dogs, 7 dogs in the heterogeneous group and 5 Beagle dogs in the homogeneous group, were electrodiagnostically examined to determine MUNE in the cranial tibial muscle, and in all 12 dogs MUNE could be elicited. Mean MUNE, CMAP, and SMUP values of the heterogeneous group were 36 ± 12, 13.86 ± 3.7 mV, and 412 ± 20 μV, respectively (Figures 2A–C). Mean values for MUNE, CMAP, and SMUP of the homogeneous group were 28 ± 4, 11.74 ± 1.69 mV, and 432 ± 40 μV, respectively (Figures 2A–C). The number of motor units within both groups did not differ significantly from each other (*P* > 0.05). Within the heterogeneous group, a significant (*P* < 0.0001) correlation between the size of CMAP amplitude and the estimated number of motor units was observed (Figure 3A), whereas in the homogeneous group no significant (*P* > 0.05) correlation between these two variables could be detected (Figure 3B). Concerning the correlation between the average size of SMUP amplitude and the estimated number of motor units, no significance (*P* > 0.05) was found in either group (Figures 4A,B). Table 2 summarizes the values for MUNE, CMAP, and mean SMUP for each dog.

**Table 2:** **Summary of electrophysiological results of each dog**.

Dog number	Number of motor units	CMAP (mV)	Mean SMUP (mV)
1	35	12.68	0.3662
2	106	34.96	0.3291
3	35	14.75	0.4174
4	14	5.42	0.3810
5	29	14.55	0.5000
6	18	6.94	0.3833
7	15	7.71	0.5095
8	43	18.47	0.4324
9	20	10.44	0.5150
10	19	10.16	0.5459
11	28	9.50	0.3421
12	31	10.11	0.3262

*CMAP, compound muscle action potential; SMUP, single motor unit potential*.

## Discussion

In the current study, we evaluated the potential use of the MUNE method for diagnostic and prognostic evaluation of canine neurological patients. All 12 dogs were successfully examined using the incremental stimulation technique. In Table 2, all values obtained in the two groups of dogs are summarized and could be used for comparison to diseased dogs with suspected loss of ventral horn cells of the spinal cord in future studies. Nevertheless, there are some common technical problems that are worth mentioning. Inherent to all electrodiagnostic procedures, artifacts from electrical noise may considerably affect the examination (4). It is, therefore, highly recommended to perform MUNE within a screened room or to apply correct filters (14). Area under the curve or amplitude measurements can be evaluated for the assessment of CMAP and SMUPs size. Area calculation includes the width of the CMAP and, therefore, more adequately reflects contribution of slower conducting motor axons (15). Hence, area calculation is considered to be a more stable factor (4). Mancuso et al. have suggested to only accept increases in the amplitude >50 μV in order to overcome the problem of alternation (8). This approach provided different heights of the average SMUP amplitudes of each dog (Table 2). When obtaining the sample of 10 SMUPs, we generally applied single pulse stimulation with constantly increasing intensity. The resulting size of amplitude of a single motor unit was either less than a 50 μV increase compared to the one evoked before, or it was far above 50 μV (e.g., 300 μV). In the first case, the stimulation intensity was further increased in order to receive an appropriate response. In the latter case, however, we observed that repeatedly applied single pulse stimulation without changing the intensity leads to varying sizes of amplitude of a single motor unit. Accepting the SMUPs, regardless of their amplitude, results in higher values for the mean SMUP. Thus, the number of motor units might be misleadingly underestimated.

It is, therefore, crucial to always attempt to record the smallest, reproducible SMUP possible, thereby meeting the demands of Mancuso et al. (8), by constantly trying to gradually increase the intensity in very small steps. Another, more general difficulty of MUNE becomes evident when the diverse mean values for the SMUPs are scrutinized. The number of motor units within a muscle is not a discrete value that can be assessed directly. It rather is the result of a ratio, including the parameters CMAP and mean SMUP. For this reason, even small changes in one of these two parameters may have a significant influence on the estimated number of motor units within the muscle of interest. The CMAP, which represents the numerator, is considerably affected by the position of the electrodes. When placed incorrectly, the number of motor units will be underestimated. Therefore, only experienced examiners should perform the measurements. In the course of a neuromuscular disease, the CMAP amplitude declines due to an acute axonal loss, resulting in lower MUNE values (4). On the other hand, the denominator (mean SMUP) reveals the average size of a SMUP. During chronic denervation, a compensatory effect, termed collateral reinnervation, may occur by sprouting of new collaterals from surviving motor axons (16). In consequence, the size of individual motor units increases (17), resulting in higher values for the mean SMUP. Thus, the decrease in MUNE within a muscle is attributable to either a decline in CMAP amplitude or an increase in the mean SMUP amplitude or both. It is, therefore, crucial to make the measurements in the most standardized way in order to avoid an over- or underestimation of the number of motor units.

Although we did not find significant differences in mean CMAP, SMUP, and MUNE values in both groups (Figures 2A–C), Table 2 depicts that the individual values do vary from one dog to another. As a result of our findings, we raise concerns about the reproducibility of MUNE and must, therefore, question to what extent these values can be compared. In the study by Simmons et al. (18), the reproducibility of MUNE in individual subjects is critically discussed. Taking previous studies into consideration, the authors stated that MUNE generally provides appropriate reproducibility for grouped data. Evaluating individual patients, however, they harbor doubts about the validity. Even if MUNE is performed twice in the same individual, values may differ from each other (18). The varying CMAP amplitudes in the heterogeneous group of dogs in the current study might be due to the different breeds, and, therefore, different sizes of the animals, but is more likely a result of the needle position. Since the majority of the dogs were client-owned, we did not want to prolong the dogs’ general anesthetic for unnecessary reasons. Therefore, we performed MUNE just once on each dog. However, in order to ensure that the MUNE values are reproducible, the examination should be performed multiple times on an individual subject, and future should particularly focus on the intraindividual variability.

The number of motor units is clearly dependent on the CMAP size (Figure 3). In the heterogeneous group, a significant correlation between these two parameters could be shown (Figure 3A). We did, as expected, not find any significant correlation between MUNE and mean SMUP in either group since we only examined neurologically healthy individuals. It is debatable whether the sole examination of the CMAP amplitude provides sufficient information. This may be the case for acute processes where no collateral reinnervation is present, provided that the examiner has the required expertise to avoid deviations caused by incorrect placement of electrodes. However, as soon as reinnervation processes occur (i.e., in chronic neurogenic conditions) CMAP may no longer adequately monitor motor unit loss. Alternatively, a prospective study by Maathuis et al. in 2010 (19) exemplified the CMAP scan and the CMAP scan-based progression score. The method described assesses axonal loss, reinnervation, and the remaining number of motor units without obtaining the mean SMUP. Hence, it avoids sample bias due to the fact that the CMAP represents all motor units firing together (6, 19). Moreover, a significant correlation between the values of CMAP scan-based progression score and MUNE was shown (19).

Although there are alternative approaches to assess axonal loss in diseases affecting the motor system and even though the perfect MUNE method has not been found yet (10), it is a well-described technique that has proven to be a sensitive marker of disease progression in motor neuron diseases (20). Therefore, MUNE should gain more attention in veterinary science. MUNE is, for instance, particularly useful for longitudinal monitoring of lower motor neuron signs in dogs affected by degenerative myelopathy as proposed by Vasquez et al. (2). This technique might, moreover, reveal useful information about canine patients suffering from the intermediate or chronic type of hereditary canine spinal muscular atrophy. This disease selectively affects motor neurons, and whereas dogs expressing the accelerated phenotype are euthanized quite early in their lives, those showing the intermediate or chronic form might cope with it for months or even years (21). Thus, MUNE might provide insight into disease progression contributing to the prognostic evaluation of those patients. Furthermore, intervertebral disk degeneration is common in canine neurology. Of particular concern is the intervertebral disk extrusion representing the most common spinal neurological disorder in dogs (22). It mostly occurs in chondrodystrophic breeds (22) and may cause a profound trauma of the spinal cord possibly leading to pain, sensory, and motor deficits (23). In some cases, it is not obvious whether to start a conservative therapy or to perform surgery, particularly in those patients that are suspected to sustain long-term deficits (23). Hence, those dogs always challenge the veterinarian as well as the owner due to the fact that it may be extremely difficult to comment on the patient’s prognosis. In a prospective study from China, researchers performed MUNE of the human tibialis anterior muscle in spinal cord injury. They reported that MUNE might indicate functional motor unit loss or transsynaptic degeneration distal to the site of the spinal cord injury (17). Therefore, MUNE appears to be an eligible technique for the diagnostic and prognostic evaluation of canine spinal cord injury patients showing lower motor neuron lesions.

In conclusion, this study does not finally settle the important question of whether MUNE represents a reliable diagnostic and prognostic parameter for the evaluation of canine neurologic patients. We have shown that it is applicable to dogs and we have pointed out its possible impact on prognostic evaluation of neurologic patients due to its ability to provide insight into the disease progression. Nevertheless, the current study was performed on dogs with no neurological diseases that were expected to interfere with the results of electrodiagnostic testing. Therefore, future studies are encouraged, including patients suffering from neurological diseases with suspected loss of ventral horn cells, to study the meaning of MUNE in canine neurological conditions. Well-designed prospective trials, including large patient groups, should particularly focus on test–retest reliability in order to establish a reliable reference range for different breeds. Comparative clinical trials of diseased and healthy individuals may ultimately answer the question about the utility of MUNE in canine neurological patients. While MUNE appears to be a promising technique in canine neurology, it will most likely not replace conventional electrophysiological techniques since the number of motor units is only estimated. Because of the described difficulties in performing this technique, MUNE will most probably not be performed routinely during the diagnostic workup, but it might gain attention in research of certain diseases, especially as a marker of disease progression in controlled follow-up studies.

## Author Contributions

JK: planning the study design, organization of electrodiagnostic testing, electrodiagnostic testing itself, including analysis of results, statistical analysis, and writing the manuscript. SP: providing expertise in the field of MUNE (human patients and rodent models), providing the EMG machine with the appropriate program, and final approval of version to be published. AT: planning the study design, organization of electrodiagnostic testing, support during electrodiagnostic testing, helping to interpret the data, review of manuscript, and final approval of version to be published. VS: planning the study design, organization of electrodiagnostic testing, support during electrodiagnostic testing, helping to interpret the data, review of manuscript, and final approval of version to be published.

## Conflict of Interest Statement

The authors declare that the research was conducted in the absence of any commercial or financial relationships that could be construed as a potential conflict of interest.
